# Cure of Nævi by Croton Oil

**Published:** 1845-02

**Authors:** 


*Cure of Nævi by Croton Oil.* By M. Lafargue.—Five or six punctures should be made on and around the tumor, with a lancet dipped in the oil, just as in vaccination. Each of the punctures causes immediately a pimple, which in thirty-six hours is developed into a little boil. These boils unite, and form a red, hot, painful tumor, covered with white crusts, and resembling a small carbuncle. Two days afterwards the scars separate, and in lieu of the nævus is seen an ulcer which is to be treated on general principles. It would be dangerous to make more than six punctures on a very young infant, as the irritation and fever are considerable.

*London and Edinburg Monthly Journal.*

